# Regulatory Cell Therapy in Organ Transplantation: Achievements and Open Questions

**DOI:** 10.3389/fimmu.2021.641596

**Published:** 2021-02-23

**Authors:** Marta Fortunato, Konstantina Morali, Laura Passeri, Silvia Gregori

**Affiliations:** ^1^Mechanisms of Peripheral Tolerance Unit, San Raffaele Telethon Institute for Gene Therapy (SR-Tiget), IRCCS San Raffaele Scientific Institute, Milan, Italy; ^2^Vita-Salute San Raffaele University, Milan, Italy

**Keywords:** regulatory T cells, tolerogenic dendritic cells, myeloid regulatory cells, cell therapy, solid organ transplantation

## Abstract

The effective development of innovative surgical applications and immunosuppressive agents have improved remarkable advancements in solid organ transplantation. Despite these improvements led to prevent acute rejection and to promote short-term graft survival, the toxicity of long-term immunosuppression regiments has been associated to organ failure or chronic graft rejection. The graft acceptance is determined by the balance between the regulatory and the alloreactive arm of the immune system. Hence, enhance regulatory cells leading to immune tolerance would be the solution to improve long-term allograft survival which, by reducing the overall immunosuppression, will provide transplanted patients with a better quality of life. Regulatory T cells (Tregs), and regulatory myeloid cells (MRCs), including regulatory macrophages and tolerogenic dendritic cells, are promising cell populations for restoring tolerance. Thus, in the last decade efforts have been dedicated to apply regulatory cell-based therapy to improve the successful rate of organ transplantation and to promote allogeneic tolerance. More recently, this approach has been translated into clinical application. The aim of this review is to summarize and discuss results on regulatory cell-based strategies, focusing on Tregs and MRCs, in terms of safety, feasibility, and efficacy in clinical studies of organ transplantation.

## Introduction

Solid organ transplantation (SOT) is a life-saving treatment for patients with end-organ dysfunction. Thanks to advances in the surgical techniques and in the use of effective immunosuppressive drugs, acute transplant rejection has been declined. Unfortunately, toxicity of immunosuppressive regimens and chronic rejection remain the main limiting factors for organ acceptance and patient survival (1). Current research focused on preventing the activation of the alloreactive responses and inducing immune tolerance (2, 3).

In the last two decades adoptive transfer of regulatory T cells (Tregs), regulatory myeloid cells (MRCs) or mesenchymal stromal cells, has become one of the most promising approach to promote/restore immunological tolerance. In the context of SOT these cell-based approaches in pre-clinical animal models demonstrated their ability to modulate alloreactive immune responses, to prevent organ rejection, and to promote long-term tolerance (4–6). These results prompted the development of protocols to expand or generate regulatory cell products for clinical application in allogeneic transplantation with the aim at preventing/modulating graft vs. host disease (GvHD) or organ rejection and at promoting tolerance. Results demonstrated the feasibility, safety, and efficacy of several regulatory cell products. An overview on tested cell-based strategies and future perspectives in SOT will be presented.

## Treg Cell-Based Therapy in Organ Transplantation

The aim of Treg cell-based therapy is to promote tolerance without interfering with the normal function of the immune system. In pre-clinical models, administration of Tregs have been used to control GvHD and organ rejection (1, 7, 8). The development of good-manufacturing-practice (GMP)-compliance protocols to isolate and expand human Tregs *ex vivo* and to generate donor-specific Tregs allowed the translation of the two main subsets of Tregs, the Forkhead box P3-expressing Tregs (FOXP3^+^ Tregs) (9) or the IL-10-producing T regulatory type 1 (Tr1) cells (10), in to clinical testing.

### *Ex vivo* Isolated, Expanded, or Induced Tregs in Allogeneic Transplantation

**Figure 1:**
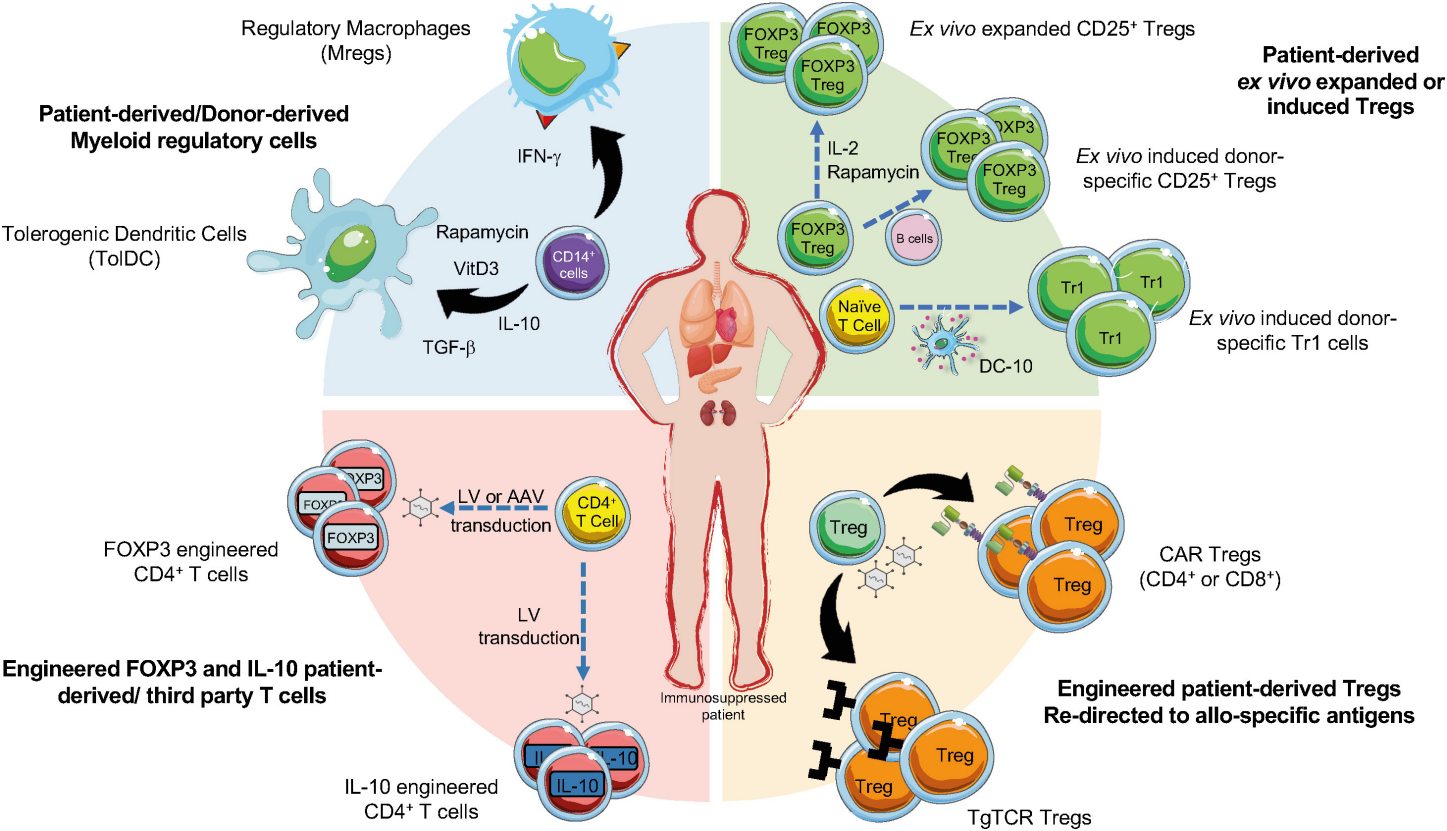
Current cell-based strategies in organ transplantation. Regulatory T cells (Tregs), regulatory myeloid cells (MRCs), and engineered Tregs have been applied as cell-based therapy to promote tolerance in pharmacological immunosuppressed patients undergoing organ transplantation. *Ex vivo* expanded Tregs can be generated in presence of different agents (e.g., IL-2, or Rapamycin). Donor-specific Tregs can be generated upon activation with CD40L-activated B cells and then expanded, CD4^+^ T cells co-cultured with allogeneic DC-10 differentiate into allo-specific Tr1 cells (green sector). Allo-specific redirected Tregs can be induced through the transduction with CARs or transgenic TCR (orange sector). The production of engineered FOXP3 and IL-10 overexpressing Tregs can be obtained by the transduction of CD4^+^ T cells with lentiviral vectors (LV) or adenoviral vectors (AAV) encoding for IL-10 or FOXP3 (red sector). MRCs, tolerogenic DC (TolDC) or regulatory macrophages (Mregs), are differentiated from CD14^+^ cells through exposure to immunomodulatory agents (e.g., IL-10, TGF-β, Rapamycin, Vitamin D3) (blue sector).

After the seminal work in 2009 demonstrating that adoptive transfer of *ex vivo* expanded Tregs modulated symptoms and allowed tapering immunosuppression in chronic GvHD (11), several clinical trials provided evidence of Treg effectiveness in this context (6), and prompted investigators to apply Treg cell-based therapy in the context of SOT (Figure 1) (12).

The first application of Tregs in SOT was conducted in patients undergoing living-donor liver transplantation treated with autologous Tregs cultured with irradiated donor cells in the presence of anti-CD80/86 agonists (13). This study demonstrated that Treg infusion led to taper immunosuppression starting from 6 months, with complete withdrawal achieved by 18 months. Similar studies, focused on the safety of the approach, have been then conducted using *ex vivo* induced donor-specific Tregs or *ex vivo* expanded Tregs in SOT (14). The ONE study, the first study aimed at comparing different cell products and at generating consensus on the standardization of the outcome of the trials (http://www.onestudy.org/), demonstrated that Treg administration in living-donor kidney transplanted patients is safe, and is associated to lower infectious complications compared to standard immunosuppressive treatments, but an overall similar rejection rates in the first year post-transplantation was observed (15). Beside the ONE study, a number of clinical trials with *ex vivo* expanded Tregs in SOT have been completed or are ongoing (NCT02166177; NCT02145325; NCT02088931; ISRCTN11038572; NCT01446484; NCT03284242; NCT01624077). Overall, these studies demonstrated that Treg-cell based therapy is a potentially useful therapeutic approach in recipients of organ transplantation to minimize the burden of general immunosuppression (16–20). Moreover, the safety profile of the treatment opened the possibility to improve its efficacy by tailoring immunosuppression regiment to favor Treg survival upon *in vivo* injection, or by combining Treg administration with low dose of IL-2, which supports Treg survival *in vivo* (21).

In line with pre-clinical data revealing that donor-specific Tregs better suppress alloreactive T cells than polyclonal Tregs (22), a protocol to generate donor-specific Tregs generated with CD40L-activated allogenic B cells (darTregs) has been established (23) and tested in liver transplantation (NCT02244801 and NCT02091232). Results showed that infusion of darTregs is safe and lowers the incidence of serious adverse effects related to infections after transplantation (15). Other clinical studies are ongoing to test safety and efficacy of donor-specific Tregs administration alone or in conjunction with costimulatory blockade therapy (NCT03577431 and NCT03654040). Alternatively, trials in which donor-specific Tregs are administered at different time points post-transplantation (ARTEMIS trial, NCT02474199) or at different cell doses (dELTA trial, NCT02188719) are ongoing.

Tr1 cells are phenotypically defined as memory T cells that co-express CD49b and LAG-3 (24), and suppress immune responses *via* an IL-10-mediated mechanism (25). Tr1 cells were identified in patients treated with allogenic-HSCT who developed immunological tolerance with mixed chimerism (26, 27). Several GMP compatible protocols have been established to generate human allo-specific Tr1 cells (28). Originally, allo-specific Tr1 cells, differentiated by culturing human PBMC (or purified CD4^+^ T cells) with allogeneic monocytes in the presence of exogenous IL-10 (29), prevented GvHD after haploidentical HSCT in adult patients affected by hematological malignancies, the ALT-TEN trial (30). The discovery of DC-10, a subset of monocyte-derived human DC that secrete IL-10 and express the tolerogenic molecules ILT4 and HLA-G (31), allowed the improvement of the protocol to generate allo-specific Tr1 cells leading to a population, which contains up to 15% of differentiated Tr1 cells (29) (Figure 1). A phase I trial was initiated (NCT03198234) in which the improved Tr1 cell product, termed T-allo10, generated by culturing patient-derived CD4^+^ T cells with donor-derived DC-10 in the presence of IL-10, is administered at the time of allogeneic HSCT. Thus far, results indicate that the therapy is well-tolerated, but effects on GvHD and long-term tolerance are under investigation (Roncarolo M.G., personal communication). An alternative protocol to generate a Tr1 cell product, named T10 cells, suitable for cell-based approaches in SOT, has been established by culturing donor-derived CD4^+^ T cells with patient-derived DC-10 in the presence of IL-10 (32). T10 cells have been planned to be tested in a clinical trial to prevent graft rejection after living-donor kidney transplantation (15), but they have not been tested yet. Finally, a protocol to expand DC-10-induced allo-specific Tr1 cells with stable phenotype and suppressive activity have been recently presented (Arteaga S. et al., FOCIS 2020). This protocol opens the window for establishing a Tr1 cell-based therapy in preventing allograft rejection.

### Engineering Tregs in Allogeneic Transplantation

Among various options to confer target specificity to Tregs, genetic engineering is highly appealing. Transduction of chimeric antigen receptors (CARs) (33) or synthetic T cell receptors (TCRs) (34) in Tregs have been demonstrated to be effective in pre-clinical studies *in vitro* and *in vivo* and are currently under intensive investigation.

CARs are synthetic proteins created by combining a single-chain antigen–binding domain derived from an antibody, fused to trans-membrane and intracellular signaling domains, usually encoding components of CD3ζ of a TCR and one or more costimulatory domains relevant for T cell activation (35). First developed for cancer immunotherapy, CARs demonstrated their feasibility in early pre-clinical studies in which CD4^+^FOXP3^+^ Treg specificity was redirected against antigen relevant to autoimmunity (36, 37). In the context of organ transplantation three groups developed Tregs expressing CAR targeting HLA-A2 (A2-CAR) to control alloreactive T cells after SOT. A seminal work in 2016 proved that A2-CAR expression in CD4^+^FOXP3^+^ Tregs enabled allo-specific recognition, proliferation, and preserved suppressive function *in vitro*. Despite this relatively strong CAR-mediated activation, A2-CAR Tregs retained high expression of FOXP3 without any significant induction of cytotoxic activity. In a humanized mouse model, A2-CAR Tregs prevented xeno-GvHD (38). Subsequently, other groups confirmed this approach, showing that A2-CAR Tregs suppress allo-responses better than polyclonal Tregs both *in vitro* and in humanized mouse models of A2^+^ skin xenografts (39, 40). A2-CAR Tregs controlled *de novo*, but not memory, alloreactivity in skin allograft immunocompetent recipients (41). A panel of humanized A2-CARs was then generated and tested in CD4^+^FOXP3^+^ Tregs showing different degree of CAR expression, ability to bind A2, and induction of Treg-mediated suppression *in vitro* and *in vivo* (42). CAR encoding the wild type form of CD28 was superior to all other CARs *in vitro* and *in vivo* in terms of proliferation, suppression, and delay of GvHD (43). Despite the need for optimization, early success with CAR Tregs already brought the authorization of the first-in-human clinical trial to evaluate A2-CAR Treg therapy (TX200) for the prevention of rejection following A2-mismatched kidney transplantation (https://sangamo.com) (Figure 1). CAR technology has been also applied to CD8^+^CD45RC^low/−^ Tregs, which delay allograft rejection in humanized mice (44), and are currently under clinical development for kidney transplanted patients (45) (https://www.reshape-h2020.eu/) (Figure 1). Pre-clinical results showed that A2-CAR CD8^+^ Tregs were significantly more effective than polyclonal CD8^+^ Tregs in preventing human skin transplant and xeno-GvHD in mice (46) (Figure 1).

Ectopic expression of a TCR, used to engineer T cell specificity in the field of cancer immunotherapy (47), has been applied also to Tregs. It has been reported that CD4^+^FOXP3^+^ Tregs expressing a transgenic TCR with direct allo-specificity were superior to polyclonal Tregs at prolonging heart allograft survival in mice (48, 49) (Figure 1). Although the development of human allo-specific TCR engineered Tregs has not been yet reported, this represents a promising approach because it recapitulates a more physiologic activation process, confers specificity for either extracellular or intracellular antigens, but limitations occur due to MHC restriction that implies matching of patients MHC genotype (12).

An alternative strategy to generate allo-specific Tregs, is the conversion of conventional T cells into Tregs by the overexpression of FOXP3 (50, 51). Lentiviral (LV)-mediated FOXP3 gene transfer into naïve CD4^+^ T cells lead to CD4^FOXP3^ T cells (Figure 1), with a stable phenotype, even in inflammatory conditions, and suppressive function *in vitro* and *in vivo* in several models. Moreover, CD4^FOXP3^ T cells do not affect immune responses to pathogens or tumor clearance in xeno-GvHD model (50, 52). Alternative FOXP3 over-expressing CD4^+^ T cells can be generated by the insertion of an enhancer/promoter proximal to the first coding exon of FOXP3 by passing epigenetic silencing of the gene. The edited cells exhibited a phenotype and cytokine profile superimposable to Tregs and showed strong immunosuppression *in vitro* and *in vivo* (51). Converted polyclonal CD4^+^ T cells into FOXP3^+^ Tregs can be used in the context of autoimmunity or allogeneic responses. Finally, to generate a more homogeneous population of IL-10 producing CD4^+^ T cells (CD4^IL−10^ cells) (Figure 1) an efficient protocol based on the use of LV encoding for human *IL-10* has been developed (53, 54). CD4^IL−10^ cells are phenotypically and functionally superimposable to Tr1 cells and suppress xeno-GvHD *in vivo* (54). These findings pave the way for the improvement of the adoptive cell therapy with IL-10-engineered T cells in patients undergoing SOT and HSC transplantation.

### Treg-Cell Based Therapy Conclusions and Future Perspectives

Clinical trials have proved the safety and feasibility of Treg-based therapy, and provided promising results on the ability of the treatment to taper immunosuppression and to prevent organ rejection at 1-year post-transplantation. Despite these results, several issues remain to be addressed. First, it is still to be defined the long-term safety profile related to Treg cell plasticity. Infused Tregs have indeed the potential to be destabilized in strong inflammatory conditions *in vivo*, adopting pathogenic T cell phenotype and functions, thereby possibly mediating graft rejection. Moreover, it is still an open question the overall long-lasting impact of Tregs on hampering immunity against infections and malignancies (55). Some of these questions will be addressed in ongoing phase II/III clinical trials.

Despite the promising clinical outcomes, cell isolation, manufacturing, dosing, specificity, and Treg tracking after infusion has been, so far, difficult. Moreover, *ex vivo* donor-specific Tregs or engineered Tregs compared to polyclonal expanded Tregs seems to be better; however, more investigation is needed to confirm the preliminary results. From clinical standpoint, one concern regarding the transgenic TCR is the mispairing with the endogenous TCR that can cause off-target effects. Moreover, engineered Tregs may have the risk of insertional mutagenesis due to viral transduction. These can be overcome by the development of CRISPR/Cas9 technology, which will further optimize the cell product (56, 57). Future potential application might be the combination of different engineering approaches to generate a more powerful (e.g., IL-10 or FOXP3) and allo-specific (e.g., CAR or TCR) cell product.

### MRC-Based Therapy in Organ Transplantation

Myeloid cells are involved in mediating immune responses after organ transplantation. Donor DCs migrate from the graft to lymph nodes and activate alloreactive T cells, which then migrate back to the graft to mediate rejection. Moreover, tissue-resident macrophages by secreting pro-inflammatory mediators sustain graft rejection contributing to alloreactive T cell expansion. In the tolerated graft the anti-inflammatory microenvironment allows the differentiation of MRCs that in turn promote Treg expansion or the conversion of allo-specific T cells into Tregs (58, 59). These evidences together with the development of protocols to differentiate MRCs *in vitro* prompted investigators to apply MRCs as cell-based therapy to promote tolerance in the contest of SOT (Figure 1).

A protocol to generate human regulatory macrophages (Mregs) that suppress alloreactive T cell responses *in vitro* has been established (60). Mregs convert allogeneic CD4^+^ T cells into IL-10-producing TIGIT^+^FOXP3^+^ Tregs *in vitro* and in a Mreg-treated kidney transplant recipient *in vivo* (61). After optimization of the Mreg manufacturing (62), the medicinal products Mreg_UKR has been tested to minimize immunosuppression after kidney transplantation (NCT02085629; ONEmreg12 trial), showing that Mregs, pre-operatively administered to transplant recipients, limited the number of infection-related adverse events and allowed tapering immunosuppression (15).

DC manipulation through exposure to anti-inflammatory and immunosuppressive agents have been shown to promote the differentiation of tolerogenic DC (tolDC) with the ability to modulate T cell responses and to promote Treg differentiation (63). The seminal study that led to the use of tolDC as cell therapy to prevent graft rejection showed that adoptive transfer of donor-derived tolDC prolonged heart graft survival in mice (64). After this work, several reports in pre-clinical models confirmed the ability of donor-derived tolDC alone or in combination with costimulatory blockade, or cyclophosphamide, to prevent allograft rejection (59). These results were confirmed in non-human primates (65–67). More recently, it has been shown that administration of autologous tolDC, named ATDCs, generated in the presence of low-dose GM-CSF, prevented graft rejection in pre-clinical models and in non-human primates (68). ATDCs, through the generation of a lactate-rich environment, dysregulate the aerobic glycolysis of T cells, which suppress T cell proliferation, and promote Treg expansion (69). These data paved the clinical testing of TolDC-based therapy (NCT03726307; NCT0164265, and NTC0225055). Donor-derived DCreg generated with Vitamin D3 and IL-10 (70) administered 1 week prior to transplantation prolonged renal allograft survival and attenuates anti-donor CD8^+^ memory T cell responses (71), and ATDCs infused in living kidney donor transplanted patients, demonstrated the ability to lower immunosuppression (15).

Comparison of clinical-grade tolDC generated with vitamin D3, IL-10, dexamethasone, TGFβ, or rapamycin demonstrated that all tolDC have a stable phenotype, but IL-10-modulated DC reproducibly induced suppressor Tregs (72). We and others developed IL-10-modulated DC (31, 73–75), and comparative analysis of DC-10, IL-10-modulated DC generated *in vitro* through the exposure of monocytes to IL-10 during DC differentiation (31), and IL-10-DC, monocyte-derived DC exposed to IL-10 during the last 2 days of DC differentiation (73), demonstrated that both cell types inhibited primary allogeneic T cell responses, but DC-10 were more effective in promoting allo-specific Tr1 cells *in vitro* (Gregori S. et al., personal communication). More recently, an efficient protocol to generate IL-10-producing human DC (DC^IL−10^) through the transduction of monocytes with a LV encoding for IL-10 has been established (76). DC^IL−10^ secrete supra-physiological levels of IL-10, are stable upon exposure to pro-inflammatory signals, recapitulate the tolerogenic ability of DC-10, and inhibited allogeneic T cell responses *in vivo* (76).

### MRC-Based Therapy Conclusions and Future Perspectives

MRC-based therapy represents an emerging approach on the context of SOT to taper general immunosuppression and to promote transplantation tolerance (77). Thus far, single administration of MRCs have been applied to transplanted patients; however, based on the assumption that tolDC promote tolerance via multiple mechanisms of immunomodulation, including the generation of a tolerogenic microenvironment that leads to a self-sustained tolerogenic process (78), possible multiple rounds of MRC administration may be more effective in dampening allogeneic T cell responses and in promoting allo-specific Tregs.

Despite the different methods to generate MRCs and the different models used, the common features converge in low expression of costimulatory and MHC molecules, maturation resistance, high expression of immunomodulatory molecules, modulation of T-cell responses and induction of regulatory cells. However, definition of shared markers and pathways by MRCs will help the comparison of the products and of their effects. Efforts to define guidelines, named minimum information, for MRCs (MITAP) have been recently reported, allowing some comparison between different cell products (79). Finally, in comparison with Tregs, MRCs have a limited lifespan upon *in vivo* delivery, overall lowering the risk of promoting adverse responses and long-term immunosuppression.

## Overall Conclusions

Tregs and MCRs have been tested in clinical trials, overall demonstrating the safety and feasibility of the approach but the efficacy must be further investigated. Several hurdles have been encountered by investigators in performing such clinical testing using these advance medicinal products (ATMPs) [reviewed in Trzonkowski et al. (80) Ten Brinke et al. (81)]. Some of the burden include the difficulties in implementing GMP-compliant protocols to manufacture cell products, the cumbersome legislation for running trials, and the regulatory and ethical approvals, which vary among the countries. Despite the results obtained thus far, a number of important issued remains to be defined such as the dose and schedule of cell infusion/s, the identification of the appropriate immunosuppressive regimen, and the best suited cells for given diseases. It cannot be indeed excluded that specific regulatory cell can be suitable for one particular approach or another. Another key aspect in the field of regulatory cell-based therapy is the identification of effective and informative assays to monitor efficacy and signs of unwanted activation of adverse immune responses. Results from ongoing trials focusing on precise immune-monitoring will provide the identification of biomarker of efficacy and will offer important tools for optimizing regulatory cell-based therapy to prevent organ transplant rejection and promoting long-term tolerance. In this regard, initiatives similar to that undertaken by “the ONE study” for comparing regulatory cell products in the same setting and immuno-monitoring, are highly recommended. Moreover the European Union Cooperation in Science and Technology (COST) Action BM1305, “Action to Focus and Accelerate Cell-based Tolerance inducing Therapies-A FACTT,” (A-FACTT) or Action BM1404, “European Network of Investigators Triggering Exploratory Research on Myeloid Regulatory Cells (Mye-EUNITER) by gathering expertise and investigators in the specific field of regulatory cell-based therapy enabled the creation of consensus on standard of common protocols and harmonizing guidelines for the analysis of cell products and clinical monitoring of immune responses after therapy. More recently, the INsTRuCT consortium, an Innovative Training Network (ITN) funded by the European Union H2020 Programme (https://www.instruct-h2020.eu/) established a network of European scientists, from academic and industry, designed to foster the pharmaceutical development of novel MRC-based therapies, by training the new generation of researchers in the field.

In conclusion, several efforts have been taken to advance regulatory cell-based therapy in the field of SOT and a number of additional investigations are necessary for rendering this approach routinely applicable to transplant recipients. The required patient specificity, thus far, hampered the wide application of cell-based strategies, since high level of expertise, time and money are needed. The use of third-party (unrelated to the donor or recipient) cells to generate an “off-the-shelf” cell product is a promising endpoint. The ongoing efforts will shed light on the development of innovative and effective strategies applicable to SOT, which will allow long term survival of the graft, preventing rejection.

## Author Contributions

All authors listed have made a substantial, direct and intellectual contribution to the work, and approved it for publication.

## Conflict of Interest

The authors declare that the research was conducted in the absence of any commercial or financial relationships that could be construed as a potential conflict of interest.
